# Extralobar pulmonary sequestration with a complication of torsion: A case report and literature review

**DOI:** 10.1097/MD.0000000000021104

**Published:** 2020-07-17

**Authors:** Lei Yang, Gang Yang

**Affiliations:** Department of Pediatric Surgery, West China Hospital, Sichuan University, Chengdu, Sichuan, China.

**Keywords:** child, computed tomography, pulmonary sequestration, torsion

## Abstract

**Rationale:**

Pulmonary sequestration is a congenital abnormality of the lower airway. It is characterized by a nonfunctioning mass of lung tissue that lacks normal communication with the tracheobronchial tree or pulmonary arteries and always receives its arterial blood supply from the systemic circulation. Most cases of extralobar pulmonary sequestrations (ELSs) are asymptomatic and found incidentally or in prenatal ultrasound screening.

**Patient concerns:**

A 10-year-old boy had severe chest pain and vomiting for 2 days.

**Diagnoses:**

ELS was diagnosed, and torsion of the ELS had developed as a complication.

**Interventions:**

In video-assisted thoracoscopic surgery, the ELS was resected.

**Outcomes:**

The clinical symptoms were relieved the 2nd day after surgery and did not recur over a follow-up period of 3 months.

**Lessons:**

In young patients with sudden abdominal pain or chest pain, in whom computed tomography shows a well-defined mass of homogeneous soft-tissue density in the thorax, ELS with torsion should be suspected. The presence of a feeding artery greatly supports the diagnosis of ELS, and the absence of this classic finding may indicate torsion of the pulmonary sequestration.

## 1 Introduction

In pulmonary sequestration, a nonfunctioning mass of lung tissue lacks normal communication with the tracheobronchial tree, and it always receives its arterial blood supply from the systemic circulation. Pulmonary sequestrations are divided into the extralobar pulmonary sequestration (ELS) and intralobar pulmonary sequestration according to the relationship of the aberrant segmental lung tissue to the pleura.^[1]^ The intralobar type, which account for approximately 75% of pulmonary sequestrations, is located within a normal lobe and lacks its own visceral pleura. ELSs are located outside the normal lung, have their own visceral pleura, are usually asymptomatic, and are found prenatally or incidentally.^[2]^ Symptomatic ELS caused by pedicle torsion is extremely rare, and the early recognition is difficult. We describe an unusual case of severe chest pain and vomiting caused by torsion of ELS in a 10-year-old boy.

## 2 Case presentation

**Figure 1:**
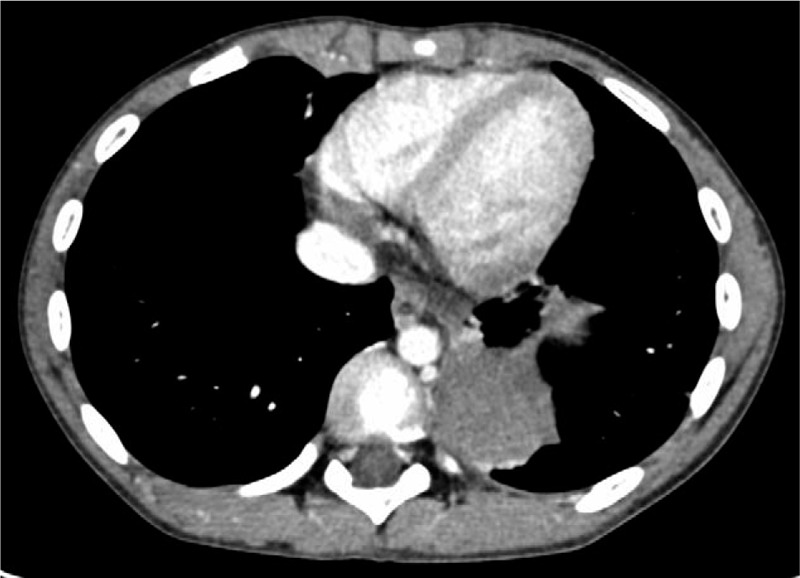
Torsion of extralobar pulmonary sequestration in a 10-year-old boy. Contrast medium-enhanced chest computed tomography showed a heterogeneous solid mass with no enhancement in the left lower thorax.

**Figure 2:**
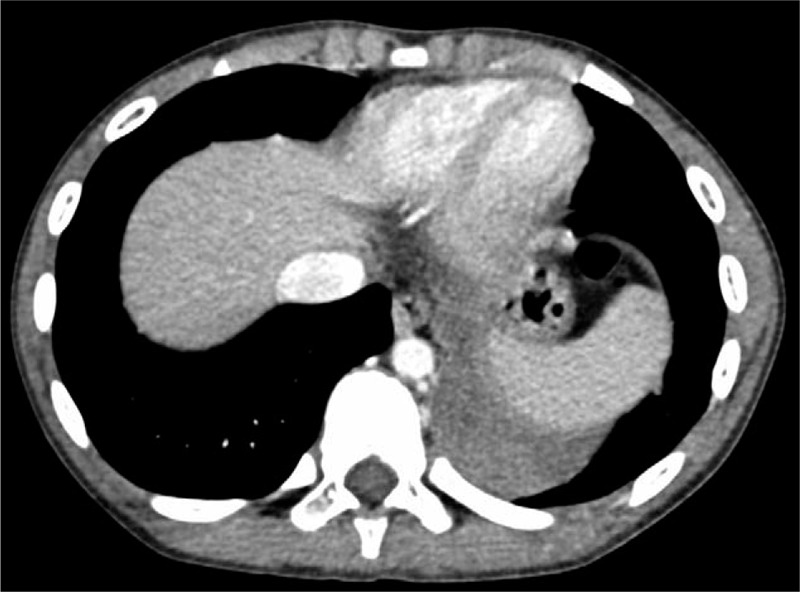
Torsion of extralobar pulmonary sequestration in a 10-year-old boy. A small amount of pleural effusion was identified on the left side.

**Figure 3:**
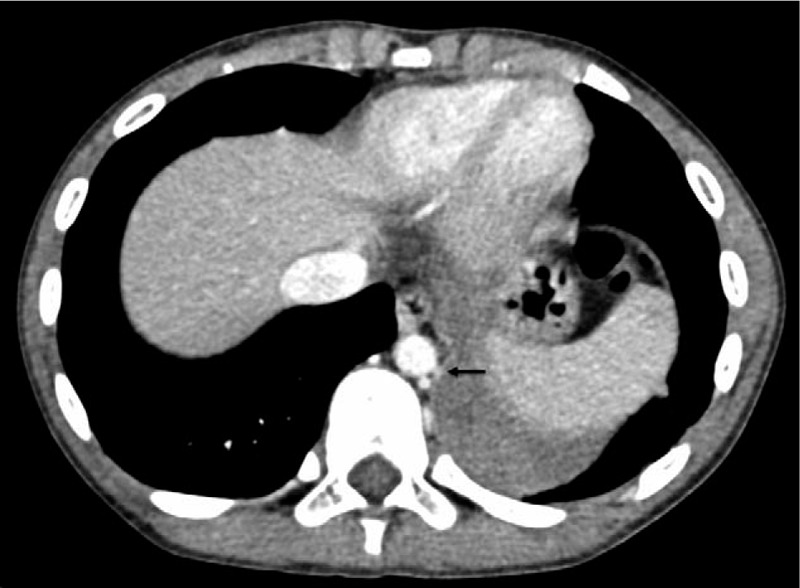
Torsion of extralobar pulmonary sequestration in a 10-year-old boy. On contrast medium-enhanced chest computed tomography, the suspected feeding artery arising from the aorta is evident (black arrow).

A 10-year-old healthy boy presented with a 2-day history of severe chest pain on the left side and vomiting, with no fever or sputum. There were no other remarkable findings in the physical examination, medical history, and family history yielded no other remarkable findings. The patient had not undergone antenatal ultrasound screening or a pre-incident imaging examination. Radiographs taken at a local hospital showed a high-density mass in the left thoracic cavity with a small amount of pleural effusion, which was suspected to be diaphragmatic hernia, and he was transferred to our center immediately. The patient still had chest pain with stable vital signs and exhibited no guarding during the abdominal examination after admission. Laboratory tests showed slight elevations in the white blood cell count (12.74 × 10^9^/L), of which the neutrophils accounted for 81.1%; the myoglobin level was 38.12 ng/mL, and other values were within normal ranges. Because radiographs were available from the local hospital and in the emergency situation, we did not perform abdominal ultrasonography. Contrast medium-enhanced computed tomography (CT) of the chest and abdomen revealed a well-defined, nonenhanced mass with soft-tissue density (3.8 × 4 × 4.7 cm) in the left thoracic cavity above the diaphragm (Fig. 1). The surrounding structures did not show any abnormalities except slight uplifting of the diaphragm and a small amount of pleural effusion on the left side (Fig. 2). On the CT scan, a suspected feeding artery arising from the aorta could be identified (Fig. 3).

Emergency video-assisted thoracoscopic surgery was performed. Intraoperative findings included the presence of a dark-brown ovoid mass (3.5 × 4 × 4.5 cm) adherent to the aortic wall and a small amount of hemorrhagic pleural effusion in the left pleural cavity. The parietal pleura near the mass appeared to be red. The pedicle that connected the mass to the mediastinum was short (1.0 cm in length), thin (0.6 cm in diameter), and twisted 180°. The blood supply to the mass arose from the thoracic aorta, and an accompanying vein drained to the intercostal vein. The mass was clipped and then resected at the pedicle. The pathological examination confirmed the diagnosis of ELS with infarction caused by torsion. The patient recovered very well and was discharged the second day after surgery. During the 3-month follow-up period, no complication or recurrence was observed on chest radiographs. The final diagnosis was an ELS infarcted as a result of torsion. (In the emergency situation, we failed to keep the preoperative chest radiograph from the local hospital and the intraoperative pictures.) This study was approved by the Human and Ethics Committee for Medical Research at Sichuan University in accordance with the Declaration of Helsinki. Written informed consent was obtained from parents of patient involved in the study.

## 3 Discussion and conclusions

Pulmonary sequestration is rare, with an estimated incidence of only 0.225% to 0.425%, and it accounts for only 0.15% to 6.4% of all congenital abnormalities of the lower respiratory tract.^[3]^ ELS is less common than the intralobar type. In the majority of cases, ELS is on the left side, between the left lower lobe and diaphragm. ELS lesions have no bronchial connection to the normal proximal airway or normal lung, and infectious complications are also uncommon. ELS is usually asymptomatic and found prenatally or incidentally in young children, torsion, and infarction of ELS are extremely rare.^[4]^

Only 11 cases of pulmonary sequestration with torsion have been reported in the literature^[5–15]^ (Table 1). In these 11 cases, ELSs were found in the thorax on the left side in 10 cases; only 1 ELS was found on the right side. These cases were found in 3 adult patients and 8 pediatric patients. Of the adults, 2 were males and 1 was female, with an average age of 29 years. The pediatric group included 5 boys and 3 girls, with an average age of 11 years (range, 6–13 years). Thus ELS with torsion seems to be more common in male patients (7 males, 3 females), and this assumption is also supported by a review of the literature, which indicates that ELS occurs more commonly in male patients, with a ratio between 3:1 and 4:1.^[16]^

**Table 1:**
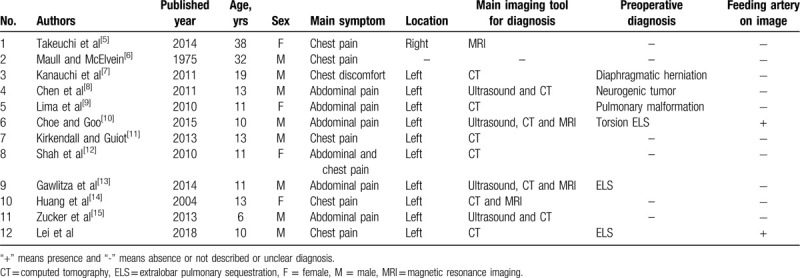
Summary of torsion extralobar pulmonary sequestration reported in literature.

All the adult patients experienced pain or discomfort mainly in the chest. Only 2 of the 8 pediatric patients had chest pain; abdominal pain was the most common symptom in the other 6. Of all 11 patients, 9 underwent unenhanced or contrast medium-enhanced CT preoperatively, and in only 1 case was a suspect feeding artery visible. Pleural effusion was present in all 11 patients. The preoperative diagnoses included diaphragmatic herniation, tumor, malformation, pulmonary sequestration, and unclear disease.

Because the initial symptoms in children were mainly abdominal pain, it was either missed or delayed. The symptoms of ELS with torsion depend on the location of the lesion and whether the children could articulate their symptoms clearly. Young children with ELS and torsion near the diaphragm on the left pleural cavity usually complain of abdominal pain, rather than chest pain. Thus, ELS with torsion should be considered in children presenting with abdominal pain and a thoracic mass on imaging, despite the fact that torsion with ELS is rare.

Imaging for diagnosis includes CT, magnetic resonance imaging, angiography, and ultrasonography.^[8]^ Angiographic appearance of a feeding artery is very strong evidence for the diagnosis of ELS. However, the most widely used tool still is unenhanced or contrast medium-enhanced CT, which were the first choices of imaging in the reported 11 cases.

The imaging features of CT include a homogeneous, well-defined mass of soft-tissue density in the thorax. Although the appearance of a feeding artery greatly supports the diagnosis of ELS,^[17]^ torsion can cause the supply artery not to appear on imaging, which causes difficulty in diagnosing ELS with torsion. Of the reported 11 patients, only 2 received the correct diagnosis preoperatively. Choe and Goo^[10]^ reported a case in which they found the feeding artery on axial T1-weighted magnetic resonance imaging, which helped establish the correct diagnosis of ELS with torsion preoperatively.

Thoracotomy or video-assisted thoracoscopic ELS resection is the standard treatment for ELS with torsion. Chest drainage is not always necessary, depending on the pleural effusion and the surgery.^[18]^

If a young patient presents with sudden abdominal pain or chest pain, and if CT shows a homogeneous, well-defined mass of soft-tissue density in the thorax, the clinicians should suspect ELS with torsion. The appearance of a feeding artery is, as mentioned, evidence of ELS, and the absence of this classic finding may indicate ELS with torsion. In addition, clinicians need to differentiate ELS from congenital pulmonary airway malformation, diaphragmatic hernia, tumor, and causes of symptoms.^[12]^

With modern imaging tools, it is easier to find the lesions; however, the preoperative diagnosis of ELS with torsion remains difficult. It is also important to identify the types of ELS in which torsion is most likely to occur; this would lead to earlier preventive ELS resection.

## Author contributions

LY wrote the first draft of the manuscript; GY performed the clinical practice. All authors reviewed the manuscript for important intellectual content and approved the final version to be published.

**Conceptualization:** Gang Yang.

**Writing – original draft:** Lei Yang.

**Writing – review & editing:** Gang Yang.
